# Prevalence and clinical pattern of paediatric HIV infection at the University College Hospital, Ibadan, Nigeria: a prospective cross-sectional study

**DOI:** 10.1186/1824-7288-37-29

**Published:** 2011-06-16

**Authors:** Babatunde O Ogunbosi, Regina E Oladokun, Biobele J Brown, Kikelomo I Osinusi

**Affiliations:** 1 Department of Paediatrics, University College Hospital, Ibadan, Nigeria; 2 Department of Paediatrics, College of Medicine, University of Ibadan, Ibadan, Nigeria

**Keywords:** HIV, Paediatric, Prevalence, Pattern, Nigeria

## Abstract

**Background:**

The prevalence of Paediatric HIV infection is largely unknown in many countries in sub-Saharan Africa. This study was aimed at determining the prevalence, clinical pattern of HIV infection and outcome among new patients aged <15 years using age-specific diagnostic methods.

**Methods:**

A prospective cross sectional study was carried out using the provider initiated HIV testing and counselling (PITC) model. HIV rapid test in parallel was used for screening and confirmation was with HIV DNA PCR in children <18 months and Western Blot in children ≥ 18 months.

**Results:**

A total of 600 children were enrolled with ages ranging between one day and 179 months. Male: female ratio was 1.2:1. HIV seroprevalence was 12.3% and after confirmatory tests, the prevalence was 10%. Fourteen (37.8%) of the children aged less 18 months were exposed but not infected. Mother-to-child transmission accounted for 93.3% of cases. Features predictive of HIV infection were diarrhoea, cough, weight loss, ear discharge generalized lymphadenopathy, presence of skin lesions, parotid swelling and oral thrush. About 75% presented in advanced or severe clinical stages of the disease, 56.8% had severe immunodeficiency while 50% had viral loads more than 100,000 copies/ml. Mortality rate was 14.3% among HIV positive compared with 11.3% in HIV negative children but was not significant. Among the HIV positive children, 26.7% were orphans.

**Conclusions:**

The prevalence rate of HIV infection among new patients screened using the PITC model was high, majority resulting from mother-to-child transmission. Most children presented in advanced stages of the disease and mortality rate among them was high. Though, the study site being a referral centre might have contributed to the high prevalence observed in this study, there is a need to expand access to PMTCT services, ensure implementation of PITC in paediatric settings and expand support services for HIV infected children.

## Background

The Human Immunodeficiency Virus (HIV) pandemic is one of the most devastating epidemics in recorded history. Though revised estimates in 2007[1] indicated substantial reduction in the estimated number of people living with HIV, sub-Saharan Africa still accounts for over two thirds (68%) of infections[1]. The global prevalence of HIV has remained unchanged at 0.8% between 2001 and 2007[1], though regional variations exist. Sub-Saharan Africa has the highest prevalence of 5.0%[1], with prevalence rates a high as 39% in KwaZulu-Natal province of South Africa[2].

Nigeria currently has the largest burden of paediatric HIV in the world, but few studies have been done on HIV in children in Nigeria. Prevalence rates in Nigeria are gotten from sentinel surveys using first time ANC attendees, the latest being (4.6%)[3] in 2007. There is however no figure for the paediatric age group[4]. Retrospective studies among high risk hospitalized Nigerian children, showed seroprevalence rates ranging from 5.7 - 20%[5]. Many employed serological assays making their application rather limited[5-8].

More than 90% of new paediatric HIV cases are acquired through mother-to-child-transmission[9]. Transfusion associated HIV infection is less common and often in settings without routine HIV screening of blood products, especially in developing countries [5,6,8].

The clinical presentation varies with the degree of immunosuppression, from asymptomatic infection to Acquired Immunodeficiency Syndrome (AIDS) characterized by severe immunosuppression and recurrent severe opportunistic infections. The predominant morbidity pattern in HIV infected children include respiratory infections, otitis media, malnutrition, diarrhoea disease, anaemia and septicaemia[5-8,10-14]. These conditions are also prevalent among children without HIV infection in most developing countries leading to a low index of suspicion for HIV infection and hence late diagnosis. The provider initiated HIV testing and counselling (PITC) has been proposed as one of the ways to increase the uptake of HIV testing and thus improve access to HIV prevention, treatment and care services, especially in areas with generalized epidemics[15]. The objectives of this study were to determine the prevalence and clinical pattern of HIV infection among children seen in the Department of Paediatrics of the University College Hospital (UCH), Ibadan using the PITC model and age-appropriate serological and virological methods.

## Methods

The study was a prospective cross-sectional study among new patients aged less than 15 years presenting at the Department of Paediatrics of UCH, Ibadan from July to December 2007. UCH Ibadan is the premier teaching hospital in Nigeria and a referral hospital for South-West Nigeria and beyond. All consecutive new patients aged less than 15 years whose parents/guardians gave written informed consent (and assent obtained for those aged 10-14 years) were enrolled into the study using the WHO guideline on PITC till the calculated sample size was exceeded. Detailed clinical information was collected using case record forms. These included demographic and socioeconomic data[16], source and reason for referral, immunization history, nutritional history, history of risk factors for HIV infection, presenting complaints, details of symptoms, any co-morbid state and a thorough physical examination.

All subjects had an initial double rapid HIV antibody tests in parallel at the point-of-care using HIV 1/2 STAT-PAK™ (by CHEMBIO DIAGNOSTICS SYSTEMS, INC. Medford, New York 11763 USA) and Determine™ HIV 1/2 (by ABBOTT JAPAN CO.,LTD. Minto-Ku, Tokyo, Japan) according to the manufactures' instructions. HIV infection was confirmed in those with a reactive rapid test by Western Blot test if aged ≥18 months and HIV DNA PCR tests for those <18 months, these were done at the Department of Virology, UCH Ibadan, a referral centre for HIV testing. Children with double rapid HIV antibody tests that were non-reactive were reported as HIV-uninfected[17,18]. Patients with HIV infection had viral load estimation, CD4 count and percentages and were enrolled at an on-going HIV/AIDS treatment programme at the study centre. Those requiring admission were admitted into the wards at the study site by the managing teams and were followed up by the study team till discharge or otherwise and the final diagnosis at discharge recorded.

Ethical approval for the study was obtained from the Joint University of Ibadan/University College Hospital Ibadan Health Research and Ethics Committee.

## Results

Six hundred patients were enrolled into the study, of which 329 (54.8%) were males and 271 (45.2%) were females, giving a male: female ratio of 1.2:1. The age of the subjects ranged from one day to 179 months with a median age of 16 months, 248(41.3%) were less than 12 months and 106 (17.7% ) were neonates (Table 1). Seventy four of the 600 children tested were reactive to the two rapid HIV test kits used, giving HIV seroprevalence rate of 12.3%. Of the 74 seropositive children, 60 were confirmed to be HIV infected, this was by HIV DNA PCR in 23 (38.3%) children aged <18 months and by Western Blot in the remaining 37 (61.7%) children aged ≥ 18 months old, giving a prevalence rate of 10.0% in the study population. Fourteen (37.8%) of the children aged <18 months were retroviral exposed but not infected as at the time of screening. In 548 children, HIV was not suspected based on their clinical features, nor were they referred specifically for HIV testing based on a prior positive testing else where or parental seropositivity. Of these, 3.1% (17/548) were seropositive and 1.8% (10/548) were later confirmed to be HIV infected using age-specific confirmatory methods.

**Table 1:** Demographic characteristics of the subjects.

*Age*	*Gender*				*Total*	
			
	Male		Female			
	
	**No**.	%	**No**.	%	**No**.	%
<12 months	134	40.7	114	42.1	248	41.3
12 - <24 months	46	7.7	42	7.0	88	14.7
24 - <36 months	21	3.5	19	3.2	40	6.7
36 - <48 months	25	4.2	19	3.2	44	7.3
48 - <60 months	22	3.7	16	2.7	38	6.3
60 - <180 months	81	24.6	61	22.5	142	23.7
**Social class**						
I	22	6.7	19	7.0	41	6.8
II	58	17.6	41	15.1	99	16.5
III	109	33.4	85	31.4	194	32.3
IV	121	37.1	104	38.4	225	37.5
V	10	3.0	17	6.3	27	4.5
Unknown	9	1.8	5	1.8	14	2.3
Total	329	54.8	271	45.2	600	100

The probable mode of transmission was mother-to-child transmission in 56 (93.3%) children, via blood transfusion in 2(3.33%), while the mode of transmission was inconclusive in 2 (3.33%). The inconclusive modes of transmission were in two sexually active adolescents who had never received blood transfusion. The first was a 13 year old girl whose mother was HIV positive and the second was a 14 year old boy from an orphanage whose mother was a deceased commercial sex worker. His elder brother who resides in the same home was seronegative.

Fever was the commonest presenting symptoms in HIV infected children, other presenting symptoms were cough, diarrhoea, weight loss/poor weight gain, and ear discharge. (Table 2). All these features were significantly more common in HIV infected patients compared with the HIV uninfected group. Also a significant proportion (p = 0.000) of HIV infected children had oral thrush, generalized lymphadenopathy, parotid fullness, otorrhoea, skin lesions, hepatomegaly and splenomegaly. Less common features among the HIV infected children were seizures, loss of consciousness and jaundice. A significant proportion of the HIV infected children were orphans (16/60) 26.7%, compared with HIV uninfected children (23/517) 4.4%.

**Table 2:** Symptoms, signs and orphan status in HIV positive and negative subjects

	*HIV Status*							
					
	Infected (n = 60)		Uninfected (n = 540)		*Total* *N = 600*		*OR (95% CI)*	*P*
			
	**No**.	%	**No**.	%	**No**.	%		
**Symptom**								
Fever	45	75.0	310	57.4	355	59.2	2.23(1.21,4.10)	0.008
Cough	39	65.0	128	23.7	167	27.8	5.98(3.39,10.53)	0.000
Fast breathing	8	1.3	82	15.2	90	15.0	0.86(0.39,1.88)	0.849
Diarrhoea	24	40.0	57	10.6	81	13.5	5.65(3.15,10.14)	0.000
Weight loss/falter	25	41.7	63	11.7	88	14.7	5.41(3.04,9.62)	0.000
Seizures	2	3.3	106	19.6	108	18.0	0.14(0.03,0.59)	0.001
Coma	-	-	21	3.9	21	3.5	-	0.254
Chronic diarrhoea	7	11.7	1	1.9	8	1.3	71.19(8.60,589.65)	0.000
Recurrent otorrohea	11	18.3	11	2.0	22	3.7	10.80(4.45,26.17)	0.000
**Signs**								
Pallor	20	33.3	150	27.8	170	28.3	1.30 (0.74,2.30)	0.368
Jaundice	2	3.3	74	13.7	76	12.7	0.22 (0.05,0.91)	0.022
Oral thrush	8	13.3	8	1.5	16	2.7	10.23 (3.69,28.4)	0.000
Lymphadenopathy	33	55.0	33	6.1	66	11.0	18.78 (10.12,34.86)	0.000
Parotid fullness	13	21.7	3	0.6	16	2.7	49.51(13.63,179.92)	0.000
Otorrhoea	7	11.7	6	1.1	13	2.2	11.76 (3.81,36.26)	0.000
Skin lesions	24	40.0	32	5.9	56	9.3	10.58 (5.65,19.83)	0.000
Hepatomegaly	43	71.7	236	43.7	279	46.5	3.26 (1.81,5.86)	0.000
Splenomegaly	19	31.7	66	12.2	85	14.2	3.33 (1.82,6.08)	0.000

Persistent fever: Unexplained fever (intermittent or constant) lasting ≥ 1 month, Chronic diarrhoea: At least two loose stools per day for ≥ 30 days, Recurrent otorrhoea: ≥ 2 episodes of ear discharge from the same ear

Symptoms predictive of HIV infection by logistic regression analysis after controlling for confounding symptoms were diarrhoea, cough, weight loss and ear discharge, with diarrhoea being the most independent predictor of HIV infection (Table 3). Signs predictive of HIV infection were generalized lymphadenopathy, presence of skin lesion, parotid swelling and oral thrush being predictive of the presence of HIV infection after controlling for other confounding signs (Table 3).

**Table 3:** Logistic regression analysis of symptoms and signs predictive of HIV infection

	*P*	*Odds ratio(OR)*	*95.0% CI forOR*
Diarrhoea	.000	0.281	0.143, 0.554
Cough	.000	0.224	0.123,0.408
Weight loss/FTT	.002	0.350	0.180,0.679
Ear discharge	.013	0.200	0.056,0.716
Generalized lymphadenopathy	0.000	0.141	0.067,0.297
Skin lesion	0.000	0.210	0.095,0.463
Parotid fullness	0.010	0.121	0.024,0.604
Oral thrush	0.026	0.223	0.060,0.834
Hepatomegaly	0.061	0.499	0.242,1.033
Splenomegaly	0.284	0.629	0.271,1.459
Otorrhoea	0.753	0.750	0.126,4.484

The Z-scores of Weight-for-length/height (WHZ), Length/height-for-age (HAZ) and Weight-for-age (WAZ) were significantly different for the HIV infected and HIV uninfected groups. The proportion on children who had Weight-for-length/height (WHZ) Z scores indicative of mild-to-moderate and severe malnutrition was similar in HIV infected and HIV uninfected children, while the proportion in children who had Length/height-for-age (HAZ) and Weight-for-age (WAZ) Z scores indicative of mild-to-moderate and severe malnutrition were significantly higher in the HIV infected children than in the HIV uninfected children. In all the parameters assessed, the proportion of the various degrees of wasting and stunting was higher among the HIV infected patients (Tables 4 and 5). None of the children in the study population had oedema.

**Table 4:** Mean Z-scores of nutritional indices among HIV positive and negative subjects

*Nutritional status (*Z-score*)*	*HIV positive (n = 60)*		*HIV negative (n = 540)*		*Z-test*	*P*
			
	*Mean*	*SD*	*Mean*	*SD*		
Weight-for-length/height (WHZ)	-1.63	2.06	-0.93	2.47	2.37	0.017
Length/height-for-age (HAZ)	-3.23	2.19	-1.86	3.55	7.60	0.000
Weight-for-age (WAZ)	-3.03	1.75	-1.79	2.13	6.26	0.000

**Table 5:** Prevalence of mild-moderate and severe malnutrition in HIV positive and negative patients

*Nutritional status*	*All subjects* *(n = 600)*	*HIV positive* *(n = 60)*	*HIV negative* *(n = 540)*	*Z-test*	*P*
	% Prevalence	% Prevalence	% Prevalence		
WHZ <-2.0	30.2	34.2	29.8	0.45	0.164
HAZ <-2.0	46.6	71.8	44.1	5.25	0.003
WAZ <-2.0	44.5	76.3	42.5	5.07	0.030
WHZ <-3.0	18.0	26.3	17.1	2.10	0.180
HAZ <-3.0	31.6	53.8	29.3	6.13	0.003
WAZ <-3.0	27.5	46.3	25.6	4.96	0.009

Associated diagnoses in the HIV infected patients were Bronchopneumonia (7/60, 11.7%), Pneumocystis jiroveci pneumonia (3/60, 5.0%), Chronic otitis media (12/60, 20.0%), Pulmonary tuberculosis (24/60, 40.0%), Tuberculous adenitis (1/60,1.7%), Acute diarrhoea disease (16/60, 26.7%), Chronic diarrhoeal disease (8/60, 13.3%), Failure to thrive (2/60, 3.3%), PEM Marasmus (44/60, 73.3%), Acute uncomplicated malaria (5/60, 8.3%), Septicaemia (7/60, 11.6%), Pyomyositis (1/60, 1.7%), Pharyngitis (1/60, 1.7%), asymptomatic (4/60, 6.7%). These were not mutually exclusive with multiple diagnoses in 58.3% of patients.

The revised WHO Paediatric Clinical Staging of HIV/AIDS was used to classify degree of immunosuppresion [19]. Most patients presented in clinical stages 3 (40.0%) and 4 (33.0%), with advanced immunodeficiency in 13.6% and severe immunodeficiency in 56.8%. The viral load of the patients ranged from 200 to 4907941 viral copies/μlitre of blood with a median of 132396 viral copies/μlitre of blood. Most of the children had viral load of more than 100 000 viral copies/μlitre of blood, with 35.7% having viral load of 100 000 - <1 000 000 copies/μlitre of blood and 23.8% with viral load of >1 000 000 viral copies/μlitre of blood (Table 6). The admission and mortality pattern among the subjects were; for HIV infected children, 70% (42/60) and 14.3% compared with HIV uninfected children of 81.7%(441/540) admitted and 11.4% mortality.

**Table 6:** Clinical Staging, immunological category and viral load pattern of HIV positive children

Clinical stage	*No(%)*
Stage1 -Asymptomatic	4(6.7)
Stage 2 - Mild	12(20.0)
Stage 3 - Advanced	24(40.0)
Stage 4 - Severe	20(33.0)
Total	60(100.0)
**Immunologic category**	
No significant Immunodeficiency	10(22.7)
Mild Immunodeficiency	3(6.8)
Advanced Immunodeficiency	6(13.6)
Severe Immunodeficiency	25(56.8)
Total	44*(100.0)
**Viral Load (copies/μlitre of blood)**	
<1000	4(9.5)
1000 - <100 000	13(31.0)
100 000 - <1 000 000	15(35.7)
>1 000 000	10(23.8)
Total	42*(100.0)

* The viral load and CD4count/% of some patients were not processed at admission due to logistic problems at the laboratory.

## Discussion

The prevalence rate of paediatric HIV infection of 10.0% in this study is lower than earlier reports from Africa [20,21] and Nigeria[5,6,22]. However, the studies from Nigeria were among high risk groups suspected to have immunosuppression and all studies were done before HIV DNA PCR testing facilities were available and used serological tests which might give a false positive results in patients aged <18 months. Other studies that reported lower prevalence rates [6,8,7] were mostly retrospective and among hospitalized children thus some cases might have been missed. Conversely, the study site being a referral site might have accounted for the high prevalence rate observed. One limitation of the study however, is in the subset of HIV exposed infants <6 weeks old and those still breast feeding. This group will need a repeat HIV DNA PCR at 3 months and 6 weeks after stopping breastfeeding due to the less than optimal sensitivity of the HIV DNA PCR in infants <6 weeks and continued exposure from breastfeeding, some of these patients might actually turn out to be HIV infected. This however was not captured in this study being cross-sectional in design. A longitudinal study would better describe this.

The Provider Initiated HIV Testing and Counselling guideline[1,15] is useful in identifying HIV infection in patient presenting to health facilities and do not have the classical symptoms and signs of the infection, thereby limiting missed opportunities for early detection and care. In this study, of such children without the classical features of HIV Infection, 17 (3.1%) children were found to be seropositive for HIV infection, and 10 (1.8%) were confirmed positive for retroviral infection. This finding gives credence to the need to offer routine screening to all children presenting in health facilities as provided in the WHO guideline[1,15]. In addition HIV infected children may present with conditions which are also seen in HIV uninfected children in the general population as highlighted in this study.

The controversy as to whether gender is a risk factor for MTCT of HIV [23,24] continues. In this study, as observed by Adejuyigbe et al[5] and Oniyangi et al[8], there was a slight female preponderance, which contrast with other reports from Africa[11,14,22,20]. MTCT accounting for 93.3% of infection in this study indicates the need to intensify efforts to get MTCT service to the large numbers of Nigerian women needing it. Transmission via blood transfusion and possible sexual route observed in this study means that as in other parts of Nigeria[5,6,8,11,22] calls for the need to improve blood transfusion services and need for paediatricians to always consider sexual mode of transmission when attending to HIV infected adolescents.

The clinical presentation of children with HIV infection in Ibadan is similar to that from other parts of Africa and Nigeria[8,11,22,25]. These symptoms and signs are also seen in HIV negative children but the Odds Ratio show the higher likelihood of HIV infected children presenting with these features and some were predictive of HIV infection on multivariate regression analysis. Their presence, singly or in combination, should heighten the index of suspicion for HIV infection in children. The rarity of neurological features in HIV infected children observed here has been reported earlier[22] and might be due to the relatively long time required for neurological features to develop.

Malnutrition, particularly marasmus, and pneumonia are prevalent clinical syndromes in HIV infected African children[5,22,20]. Malnutrition was present in 76% of children in this study. It has been recognized as an underlying contributing factor to childhood morbidity and mortality and contributes to about 56% of under-five mortalities in the developing countries. The severe malnutrition observed in HIV infected children is definitely a major factor that needs attention. The high TB-HIV co-infection rate in this study re-echo's the unholy alliance between these two conditions and the burden they pose on the health of children in our environment. Efforts to expand HIV prevention, treatment and care services must thus be matched by similar services for TB prevention and treatment.

Based on the revised WHO Paediatric Clinical Staging of HIV/AIDS[19], about 75% of the patients in this study presented in advanced clinical stages 3 and 4, and similar observations had been reported from other parts of Nigeria [11]. Also 57.8% were severely immunosuppressed which also compares with 55.7% with severe immunosuppression observed by Ugochukwu et al.[11] In addition, 50% had viral loads >100,000 copies/ml. The implication of these is that most children presented at the advanced stages of the disease associated with poor prognosis and need for antiretroviral therapy. There is thus a need to evolve methods of early identification and proper management of HIV infected children to prevent clinical and immunological deterioration, which further reiterates the need for implementing the PITC guidelines.

HIV contributes significantly to orphan rate, with rates as high as 25.75% and 10.8% in sub-Saharan Africa and Nigeria respectively[1,3]. A similarly high orphan rate among HIV positive children in this study calls for an urgent need for support services for both HIV affected and infected children bearing in mind the poor social services in these parts of the world. In addition efforts need to be intensified to reduce HIV infection and AIDS related deaths in adults by scaling up HIV prevention interventions and improved access to antiretroviral therapy as this will reduce paediatric HIV from MTCT and the number of AIDS orphans.

Mortality rate was 11.6% among admitted children in the study population, with a slightly higher rate of 14.3% among HIV positive children. Higher mortality figures of 26.1-46.3% have been reported from other parts of Nigeria[5,6,8], while in a prospective 5 year study of HIV infected Rwandan children[12], the estimated risk of death at 2 and 5 years of age was 45% and 62%, respectively and the risk of dying was 21 times higher than in uninfected children. The high mortality figures observed in these studies were in the pre-antiretroviral era and so the lower mortality rate observed in this study could have been due to the antiretroviral treatment programme in place at the study centre which facilitated the management of these patients and thus gave a better outcome.

## Conclusions

The prevalence rate of HIV infection among new patients screened using the PITC model in this study was high and majority were as a result of mother to child transmission. Some children without clinical features suggestive of immune suppression were found to be positive using the PITC model adopted in the study. Most children presented in advanced stages of the disease and mortality rate among them was high. HIV infected children need to be identified early to reduce morbidity and mortality and there is the need to expand access to PMTCT services, ensure implementation of PITC in paediatric settings and expand support services for HIV infected children.

## Competing interests

The authors declare that they have no competing interest

## Authors' contributions

BOO was involved in the conception and design, collection, analysis and interpretation of data, initial draft and review of the manuscript. RO made substantial contribution to the conception and design, interpretation of data and critical review for intellectual content, BJB contributed to the design and critical review for intellectual content and KO contributed to the conception, design and critical review for important intellectual content.

All authors read and approved the final manuscript.
